# Hemodynamic evaluation of the right portal vein in healthy dogs of different body weights

**DOI:** 10.1186/1751-0147-52-36

**Published:** 2010-05-25

**Authors:** Raquel Sartor, Maria J Mamprim, Regina F Takahira, Mariana F de Almeida

**Affiliations:** 1 Department of Animal Reproduction and Veterinary Radiology, College of Veterinary Medicine and Animal Science - FMVZ, São Paulo State University - UNESP. Distrito de Rubião Jr. s/n, Botucatu, São Paulo, Brazil. CEP 18618-000

## Abstract

**Background:**

Doppler ultrasonography is an important tool for evaluating hepatic portal hemodynamics. However, no study in dogs of different body weights, in the range encountered in routine clinical veterinary practice, has been reported. It can be difficult to obtain an ideal insonation angle when evaluating the main portal vein, so evaluation of the right portal vein branch has been described in humans as an alternative. The aim of this study was to analyze, through Doppler ultrasonography, the hemodynamics in the right portal vein branch in dogs of different body weights.

**Methods:**

Thirty normal dogs were divided in three groups by weight, in order to establish normal values for mean velocity, flow volume and portal congestion index of the right portal vein branch by means of Doppler ultrasonography.

**Results:**

In all dogs ideal insonation angles were obtained for the right portal vein branch. The average velocity was similar in the three groups, but the portal congestion index and the flow volume differed, showing that the weight of the dog can influence these values.

**Conclusion:**

Doppler ultrasonography for the evaluation of flow in the right branch of the portal vein could be a viable alternative, or complement, to examining the main vessel segment. This is especially so in those animals in which an ideal insonation angle for examination of the main portal vein is hard to obtain. In addition, the weight of the dog must be considered for the correct evaluation of the portal system hemodynamics, particularly for portal blood flow and the congestion index.

## Background

Doppler ultrasonography is a safe and effective technique for evaluation of portal vein hemodynamics in dogs. Mean velocity (Vmean), average portal blood flow volume (PBFV) and congestion index (CI) are important indicators in hepatic evaluation, mainly to detect alterations such as chronic hepatic diseases that lead to portal hypertension [1]. Hemodynamic assessment of the portal vein with Doppler ultrasound is well described as a useful tool for portosystemic shunt diagnosis, and to detect the shunted vessel origin, as portal flow velocity is increased proximal to the origin of the shunt and decreased distally [2].

Knowledge of the normal values for such variables is essential to recognize and diagnose alterations that may occur in hepatic disease. Since dogs of different breeds, sizes and weights are routinely assessed, it is important to know whether these normal values differ between dogs according to size.

In the literature, there are only reports on values for mean velocity, flow volume and portal congestion index of the portal vein in healthy, medium-sized dogs [1,3]; no study comparing these variables in animals of different body weights has been reported. We hypothesize that such values are not similar between dogs with body weights that cover the range encountered in clinical veterinary practice.

Another important factor, the insonation angle, must be considered for Doppler evaluation of portal hemodynamics. This is the angle between the ultrasonographic waves and the studied vessel. An ideal angle of 0 degrees occurs when the flow and ultrasound waves are parallel, but this is hard to obtain. A large number of authors have reported that this angle must be kept below 60°, in order to measure flow velocity with a minimal margin of error [1,3-5]. Difficulty in obtaining such an angle in the portal vein at the *porta hepatis *region is described in both dogs and humans [1,3,5,6]. However, when evaluating the human portal system, the flow velocity can be measured in the right branch of portal vein, in which smaller insonation angles can be more easily obtained, allowing a more accurate velocity measurement [6].

The aim of this study was to make Doppler flow measurements from the right intra-hepatic branch of the portal vein in order to establish the normal values for mean velocity, flow volume and portal congestion index in healthy dogs; and to detect variations between groups with different body weights.

## Materials and methods

Thirty healthy dogs, males and females, of several breeds obtained from the Veterinary Hospital, College of Veterinary Medicine and Animal Science-FMVZ, São Paulo State University-UNESP, Botucatu, São Paulo State, Brazil, were evaluated. These animals were considered healthy based on physical, hematological and biochemical analyses, as well as on abdominal ultrasonography. Initially there were 42 animals, but seven dogs could not be examined due to the breathing or the behaviour of the animal, and five dogs had abnormal values of clinical laboratory tests. The animals were divided into three groups, with ten dogs in each, according to weight range: Group A, dogs weighed ≤ 10 kg; Group B, dogs weighed 10.1-20 kg; and Group C, dogs weighed ≥ 20.1 kg.

A triplex scan ultrasonographic device (GE, Logic 3 model) was used. It had two multi-frequency transducers, convex from 3.5 to 5 MHz and linear from 6 to 10 MHz.

Dogs were deprived of food for 12 hours before the test and received dimethicone, (Dimeticolin^® ^75 mg/ml, Hipolabor Farmacêutica LTDA, Borges Sabará-MG, Brazil), orally, at a dose of 4 drops/kg, three times from the beginning of the fasting period to 20 minutes before the ultrasonographic evaluation. Dimethicone is an antiflatulent and was used to avoid intestinal gas formation. No sedatives were used.

**Figure 1:**
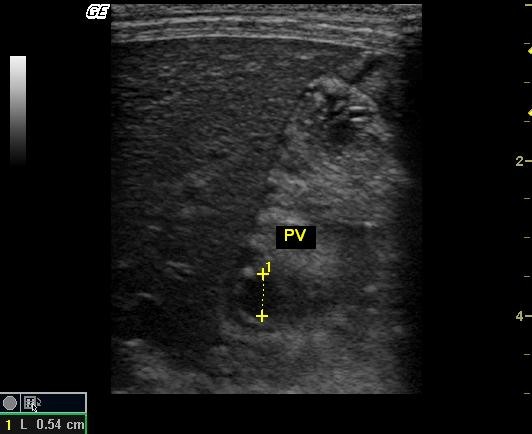
**B-mode ultrasound image**. B-mode ultrasound image showing the portal vein (PV), of one dog. Diameter measurement between callipers (0.54 cm). Transverse section at the right 11^th ^intercostals space.

Before Doppler evaluation, the diameter and area of portal vein were measured, the animal was kept in left lateral decubitus position and the transducer was placed on the right lateral body wall, at approximately the 10th or 11th intercostal space, in the *porta hepatis *region, in which the right kidney was not observed, as previously proposed by other authors [7] (Fig. 1). After diameters were determined, vessel cross-sectional areas were calculated using the following formula (1):

*A*: portal vein area; *D*: portal vein diameter; *π*: 3.14

**Figure 2:**
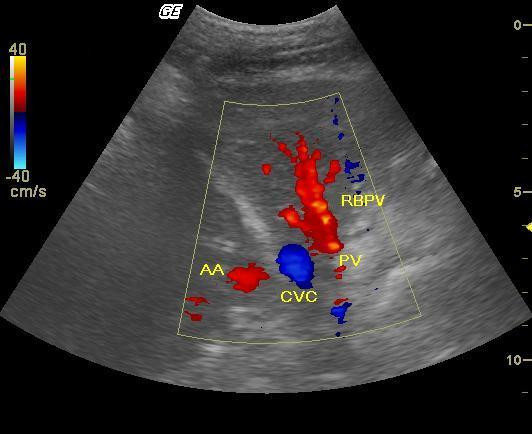
**Color Doppler**. Color Doppler mapping showing the origin of the right branch of the portal vein in one dog. Note that the axis of the vessel is very close to that of the ultrasound wave, which provides an optimal insonation angle, with the flow running towards the transducer. Longitudinal section at approximately the right 10^th ^intercostal space (RBPV: right branch of portal vein; CVC: caudal vena cava; AA: abdominal aorta; PV: portal vein).

Color and spectral Doppler examination was carried out in the right branch of the portal vein to evaluate blood flow direction and velocity within the vessel (Fig. 2). To access the right branch, the transducer was kept in the previously described position to allow the determination of the portal vein diameter [6] and was moved approximately one intercostal space cranially and the angle was set to obtain longitudinal image of the portal vein right branch, as suggested in literature [7]. The angle between the sound waves and the flow direction in the evaluated vessel was kept less than or equal to 60 degrees in all cases. Mean velocity (Vmean) was assessed through the uniform insonation technique by using the software from the device; angle correction was used in all cases to calculate the velocities accurately.

The experimental design was completely randomized, with three groups and ten replicates per group. For each animal, all measurements had three replicates, from which a mean was calculated. Based on the obtained data, portal CI and average portal blood flow volume (PBFV) were assessed.

To calculate CI, the following formula was used [8]:

*A*: portal vein area; *Vmean*: portal vein flow mean velocity

To calculate PBFV (ml/min/kg), the following formula was used (5):

*w*: body weight

It was necessary to change the unit of the Vmean (cm/sec to cm/min).

### Statistical analysis

Data were analyzed through an F-test and means compared by Tukey's test, at 5% significance.

## Results

This study included dogs of various body conformation, breed and size. In all evaluated animals, blood flow evaluation was possible in the portal right branch it was easily found, and the insonation angle was always lower than 60° (average and standard deviation 32 and 12 degrees, respectively).

Flow velocity was similar among all three groups (P > 0.05). Portal blood flow volume was significantly (P < 0.05) greater in Group A, relative to Group C, whereas the results of Groups B and C were similar. With respect to the portal CI, Group A had significantly (P < 0.01) lower values, relative to the other two groups (Table 1).

**Table 1:** Means (± SD) Doppler variables of the Right Intra-Hepatic Portal Vein.

WEIGHT (kg)	Vmean (cm/s)	PBFV (ml/min/kg)	CI (cm.s)
**≤ 10.0**	16.95 ± 5.79	51.37 ± 20.55	0.022 ± 0.01
**10.1 - 20.0**	16.98 ± 3.04	38.28 ± 8.15	0.039 ± 0.009
**≥ 20.1**	17.39 ± 4.77	32.19 ± 13.23	0.043 ± 0.009

Description of the means (± SD) variables obtained from evaluation through Duplex Doppler Ultrasonography of Portal Vein, according to dog groups. Flow Mean Velocity (Vmean); Portal Blood Flow Volume (PBFV); Portal Congestion Index (CI).

## Discussion

In human medicine, introduction of Doppler ultrasonography is considered a milestone in the diagnosis of portal hypertension. Nowadays, it is part of the initial examination of such patients, since it is considered extremely important for the diagnosis and prognosis [6].

In veterinary medicine, the Doppler technique has aided the diagnosis of hepatopathies mainly related to vascular alterations such as intra- and extrahepatic portosystemic disorders [4]. However, correlations between vascular alterations and clinical signs are not completely established. Normal values and variations between dogs of different sizes are needed as the normal values described in literature are based on studies involving medium-sized animals only [1,3].

**Figure 3:**
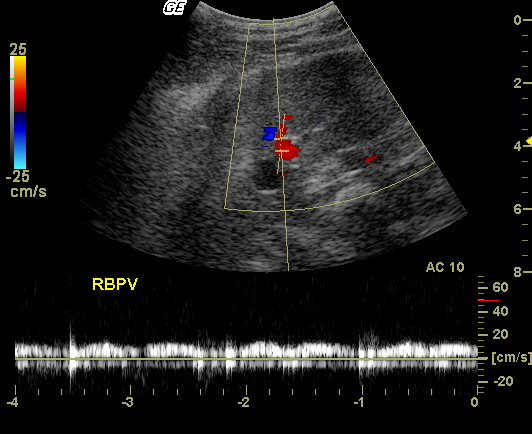
**Duplex Doppler Ultrasound**. Spectral Doppler mapping of the right branch of the portal vein (RBPV) of one dog. Longitudinal section at approximately the right 10^th ^intercostal space. Note the insonation angle (12 degrees), which provides measurements with minimum margin of error. The flow is monophasic and presents low pulsatility.

The correct insonation angle is essential for the accurate assessment of flow velocity. It has been described that in dogs with chronic hepatic diseases there is a decrease in the mean velocity of portal blood flow and this may result in clinically important portal hypertension [1]. Congenital portosystemic shunts increase the mean velocity of the portal blood flow [9]. However, insonation angles greater than 60° can produce erroneous results. This study has shown that, as described for humans [6], smaller insonation angles can be more easily obtained in the right branch of the portal vein, making its use appropriate for flow velocity measurement in dogs, with more accurate results (Fig. 3).

In this work, portal flow velocity did not differ among groups. The mean velocity values obtained in this study for portal vein right branch flow were similar to those obtained in other studies of the main portal vein, in which the normal value was between 14.7 ± 2.5 cm/s and 18.1 ± 7.6 cm/s [1].

The assessment of PBFV is important, since the main portal vein is responsible for carrying, on average, 75% of the total blood received by the liver [5]. The present study showed that the right intra-hepatic portal vein flow changes according to the weight of the dog; small-sized dogs had greater portal blood flow per kilogram body weight, than larger-sized dogs. Comparing with the results of the literature [1], our data indicate that dogs weighing ≤ 10 kg (Group A) have values above the normal range given for the main portal vein (31.2 ± 9.8 ml/min/kg) by other authors.

Measurement of CI is useful since this index increases in dogs with chronic hepatic disorders and is also considered useful in the early detection of such diseases [1]. In the present study, there was an association between body weight and this variable. In comparison with the CI values established in literature for the main portal vein, dogs from Group A had values lower than those of the previously reported normality ranges of 0.041 ± 0.018 cm.s [1] and 0.046 ± 0.012 cm.s [3], whereas the Groups B and C had values similar to the reported ones. The observed differences can be explained by the formula used for the calculation of this index:

Thus, this study demonstrated that the portal vein area in small-sized dogs is significantly smaller than that of the larger animals, while mean velocity is similar among dogs of any body weight.

This new data shows values of normality for the right portal vein flow in healthy dogs, and also an important aspect, PBFV and CI were influenced by the body weight. There is reason to believe that also the flow of the main portal vein varies with the size of the dog, and thus the reference values found in the literature [1,3] may introduce an error, since they are based only on values in medium-sized dogs.

## Conclusions

Doppler ultrasonography for the evaluation of flow in the right branch of the portal vein can be a viable alternative, or complement, to examining the main vessel segment, particularly in animals in which an ideal insonation angle for examination of the main portal vein is hard to obtain. In addition, in order to avoid misinterpretation the examiner should be aware that Doppler parameters of the portal system in dogs, mainly the PBFV and CI, are influenced by body weight.

## Competing interests

The authors declare that they have no competing interests.

## Authors' contributions

RS Principal Author of the Article (Thesis of Master Degree). Carried out the ultrasonography study, analysed the data, and drafted the manuscript. MJM Supervisor of the Master Degree Thesis (Raquel Sartor). MJM participated in the analysis of the data and in the draft of the manuscript. RKT carried out the blood analysis and the interpretation of the results. MFA auxiliary on the data collect. All authors read and approved the final manuscript.

## References

[B1] Nyland TG Fisher PE Evaluation of experimentally induced canine hepatic cirrhosis using duplex Doppler ultrasound Vet Radiol 1990 31 189 194 10.1111/j.1740-8261.1990.tb01809.x

[B2] Carvalho CF Cerri GG Chammas MC Dopplervelocimetric evaluation of portal vein as a diagnostic tool for portosystemic shunt diagnosis in dogs Ciencia Rural 2009 39 1433 1437

[B3] Lamb CR Mahoney PN Comparison of three methods for calculating portal blood flow velocity in dogs using duplex-Doppler ultrasonography Vet Radiol Ultrasound 1994 35 190 194 10.1111/j.1740-8261.1994.tb01591.x

[B4] Nyland TG Mattoon JS Diagnostic Ultrasound 2002 2 Philadelphia: Saunders

[B5] Kantrowitz BM Nyland TG Fisher P Estimation of portal blood flow using duplex real-time and pulsed Doppler ultrasound imaging in the dog Vet Radiol 1989 30 222 226 10.1111/j.1740-8261.1989.tb00777.x

[B6] Sugimoto H Kaneko T Soichiro I Takeda S Nakao A Simultaneous Doppler measurement of portalvenous peak velocity, hepatic arterial peak velocity, and splenic arterial pulsatility inex for assessment of hepatic circulation Hepato-Gastroenterol 2002 49 793 797 12063992

[B7] Szatmári V Rothuizen J Voorhout G Standard planes for ultrasonographic examination of the portal system in dogs JAVMA 2004 224 713 716 15002810 10.2460/javma.2004.224.713

[B8] Moriyasu F Nishida O Ban N Nakamura T Sakai M Miyake T Uchino H "Congestion index" of the portal vein AJR 1986 46 735 739 10.2214/ajr.146.4.735 3485345

[B9] D' Anjou MA The sonographic search for portosystemic shunts Clin Tech Small Anim Pract 2007 22 104 114 10.1053/j.ctsap.2007.05.004 17844816

